# The Seasonal Divergence in the Weakening Relationship between Interannual Temperature Changes and Northern Boreal Vegetation Activity

**DOI:** 10.3390/plants12132447

**Published:** 2023-06-25

**Authors:** Haijiang Zhao, Ning Jin, Xiurong Wang, Guiqin Fu, Kunlun Xiang, Liang Wang, Jie Zhao

**Affiliations:** 1 China Meteorological Administration Xiong’an Atmospheric Boundary Layer Key Laboratory, Xiong’an New Area 071800, China; hjzhao602@163.com; 2 Key Laboratory of Meteorology and Ecological Environment of Hebei Province, Shijiazhuang 050021, China; 3 Zhangjiakou Meteorological Bureau of Hebei Province, Zhangjiakou 075000, China; 4 Department of Resources and Environmental Engineering, Shanxi Institute of Energy, Jinzhong 030600, China; jinn.13b@igsnrr.ac.cn; 5 Public Meteorological Service Center, China Meteorological Administration, Beijing 100081, China; wangxr@cma.gov.cn; 6 Hebei Meteorological Service Center, Shijiazhuang 050021, China; fgq84@tom.com; 7 Guangdong Ecological Meteorology Center, Guangzhou 510275, China; xiangklun@mail2.sysu.edu.cn; 8 Shandong Provincial Key Laboratory of Water and Soil Conservation and Environmental Protection, College of Resources and Environment, Linyi University, Linyi 273300, China; wangliang.cn@163.com; 9 College of Natural Resources and Environment, Northwest A&F University, Yangling 712100, China

**Keywords:** temperature factors, vegetation activity, diurnal temperature range, boreal vegetation, climate change

## Abstract

The response of boreal vegetation to global warming has shown a weakening trend over the last three decades. However, in previous studies, models of vegetation activity responses to temperature change have often only considered changes in the mean daily temperature (T_mean_), with the diurnal temperature range (DTR) being neglected. The goal of this study was to evaluate the temporal trends of the relationships between two temperature factors (T_mean_ and DTR) and the vegetation activity across the boreal regions on both annual and seasonal timescales, by simultaneously employing satellite and climate datasets. We found that the interannual partial correlation between the growing season (GS) NDVI and T_mean_ (R_NDVI−Tmean_) has shown a significant decreasing trend over the last 34 years. At the seasonal scale, the R_NDVI−Tmean_ showed a significant upward trend in the spring, while in the summer and autumn, the R_NDVI−Tmean_ exhibited a significant downward trend. The temporal trend characteristics of the partial correlation between the NDVI and DTR (R_NDVI−DTR_), at both the GS and seasonal scales, were fully consistent with the R_NDVI−Tmean_. The area with a significant decrease in the GS R_NDVI−Tmean_ and R_NDVI−DTR_ accounted for approximately 44.4% and 41.2% of the boreal region with the 17-year moving window, respectively. In stark contrast, the area exhibiting a significant increasing trend in the GS R_NDVI−Tmean_ and R_NDVI−DTR_ accounted for only approximately 22.3% and 25.8% of the boreal region with the 17-year moving window, respectively. With respect to the seasonal patterns of the R_NDVI−Tmean_ and R_NDVI−DTR_, the area with a significant upward trend in the spring was greater than that with a significant downward trend. Nevertheless, more areas had a significant downward trend in the R_NDVI−Tmean_ and R_NDVI−DTR_ in summer and autumn than a significant upward trend. Overall, our research reveals a weakening trend in the impact of temperature on the vegetation activity in the boreal regions and contributes to a deeper understanding of the vegetation response to global warming.

## 1. Introduction

Boreal forests are the largest terrestrial biome in the world and store more than 30% of the global forest carbon [1,2,3]. Recent studies investigating the responses of vegetation to climatic factors in northern boreal ecosystems [4,5,6], involving satellite-derived data and manipulative experiments, have all indicated that the vegetation productivity in northern high latitudes is sensitive to temperature change [7,8,9]. The results have shown that boreal forests are closely linked to mean daily temperature (T_mean_) and the diurnal temperature range (DTR; the difference between the daily maximum and daily minimum temperature) [10]. However, most of this research was static and therefore unable to clarify the temporal variations in the relationships between the vegetation greenness and temperature factors (e.g., maximum daily temperature (T_max_), minimum daily temperature (T_min_), and DTR) over the last few decades [11].

In fact, the relationships between temperature factors and vegetation activity may change over time due to the limitations of other environmental factors at regional and global scales [5,12]. For instance, Piao et al. [5] found that the strength of the relationship between the interannual variability in vegetation greenness and the air temperature in northern ecosystems has declined substantially over the last three decades. This decline may be related to an increase in days with extreme heat and the nonlinear response of photosynthesis to air temperature in these northern high-latitude ecosystems [5]. Zhao et al. [12] investigated the inter-annual responses of vegetation greenness to diurnal asymmetric warming at the global scale over the last 34 years. They found that the decline in the vegetation greenness response to T_max_ occurred mainly in the high latitudes of the northern hemisphere, whereas the decline in the vegetation activity response to T_min_ was primarily concentrated at low latitudes.

Although the response of vegetation activity to T_mean_, T_max_, and T_min_ throughout the last three decades has been extensively investigated, the effect of DTR on vegetation activity remains largely unknown. Previous observational analyses have shown that the land surface DTR has significantly decreased, at a rate of −0.036 K decade^−1^, over the last century [13]. Huang et al. [14] examined the impact of preseason DTR on the spring vegetation phenology in the northern hemisphere and recommend that more studies should be carried out to help understand the physiological mechanisms governing the response of the start of the growing season to the preseason DTR. Variations in the link between the DTR and boreal vegetation activity over time currently remain unclear.

The main objective of this study was to explore the temporal dynamics of the relationships between temperature factors (T_mean_ and DTR) and vegetation activity across boreal North America over the last three decades, by simultaneously employing gridded meteorological data and the Normalized Difference Vegetation Index (NDVI) obtained from National Oceanic and Atmospheric Administration (NOAA) satellites. The insights derived from the findings are expected to have important implications for studying climate change and its effect on boreal forests.

## 2. Data and Methods

### 2.1. Data Sources

The third-generation NDVI dataset for the period of 1982–2015 used in this study was produced by the Global Inventory Modelling and Mapping Studies group (GIMMS) from the NOAA/AVHRR, which has been corrected for sensor degradation, cloud cover, solar zenith angle, and viewing angle effects due to volcanic aerosols and satellite drift [15]. This dataset is the longest sequence of NDVI data released to date and has been widely used in investigations of large-scale vegetation activity and dynamics [2,16]. The biweekly NDVI data with a spatial resolution of 8 km × 8 km were further aggregated to 0.5° × 0.5° to match the resolution of the meteorological data [17,18].

The monthly T_mean_, T_max_, and T_min_ and precipitation data with a spatial resolution of 0.5° × 0.5° were compiled from the Climate Research Unit, University of East Anglia (CRU TS 4.01) [19]. This dataset was produced using angular-distance weighting interpolation [12] and has been widely used in studies on the relationship between regional and global vegetation activity and climate change [20,21,22]. The DTR was calculated as the difference between the T_max_ and T_min_ values obtained from the CRU TS 4.01 product.

### 2.2. Methods

The study covered the boreal (as defined by the Köppen–Geiger climate classification, www.gloh2o.org/koppen, accessed on 20 March 2023) vegetated areas (defined as areas with a mean growing season (GS) NDVI during 1982–2015 larger than 0.1) north of 40° N [21]. The study period, which was based on the availability of satellite observations, was 1982–2015. The GS was defined as April to October, while spring, summer, and autumn were defined as April to May, June to August, and September to October, respectively [5,21].

To determine the temporal trends in the relationships between the vegetation activity and temperature factors (T_mean_ and DTR), we first calculated the first-order partial correlation coefficients between the averaged GS NDVI, GS T_mean_ (R_NDVI−Tmean_), and DTR (R_NDVI−DTR_) for each of the 17-year moving windows from 1982–1998 to 1999–2015 (i.e., 1982–1998, 1983–1999, …, 1999–2015), with the sum of precipitation as the control variable. Specifically, the first-order partial correlation coefficient was calculated using the correlation coefficient. The correlation coefficient was calculated using Equation (1).

(1) $$r_{xy}=\frac{\sum_{i=1}^{n}\left(x_{i}-\overset{-}{x}\right)\left(y_{i}-\overset{-}{y}\right)}{\sqrt{\sum_{i=1}^{n}{\left(x_{i}-\overset{-}{x}\right)}^{2}{\left(y_{i}-\overset{-}{y}\right)}^{2}}}$$

where *x* and *y* are the variables for which the correlation coefficient needs to be calculated.

Therefore, the first-order correlation coefficient can be calculated using Equation (2).

(2) $$r_{xy\cdot a}=\frac{r_{xy}-r_{xa}r_{ya}}{\sqrt{1-r_{xa}^{2}}\sqrt{1-r_{ya}^{2}}}$$

where *r_xy_*, *r_xa_*, and *r_ya_* are the correlation coefficients and *a* is the control variable.

Accordingly, there were 18 moving windows with centers ranging from 1990 to 2007, and 18 corresponding values for the R_NDVI−Tmean_ and R_NDVI−DTR_. These values were then regressed using a unary linear regression model (Equation (3)) against the centers of the moving windows to determine their respective temporal trends [12].

(3) y=αt+β+ε

where *α* is the regression coefficient; *t* is a year in the time series; *β* is the regression constant; and *ε* is the fitted residual.

Least squares fitting was employed to determine the trends at both the inter-annual and gridded scales, with statistical significance considered at the 5% (or 1%) level.

A similar approach was used to calculate the temporal changes in the relationships between the vegetation activity and temperature during the different seasons. In addition, to ensure the robustness of the research results, the analysis was also conducted with the 15-year and 19-year sliding windows, as described above.

## 3. Results

### 3.1. Trends of Correlations between Vegetation Activity and Temperature Factors

#### 3.1.1. Inter-Annual Changes in R_NDVI−Tmean_ and R_NDVI−DTR_

**Figure 1:**
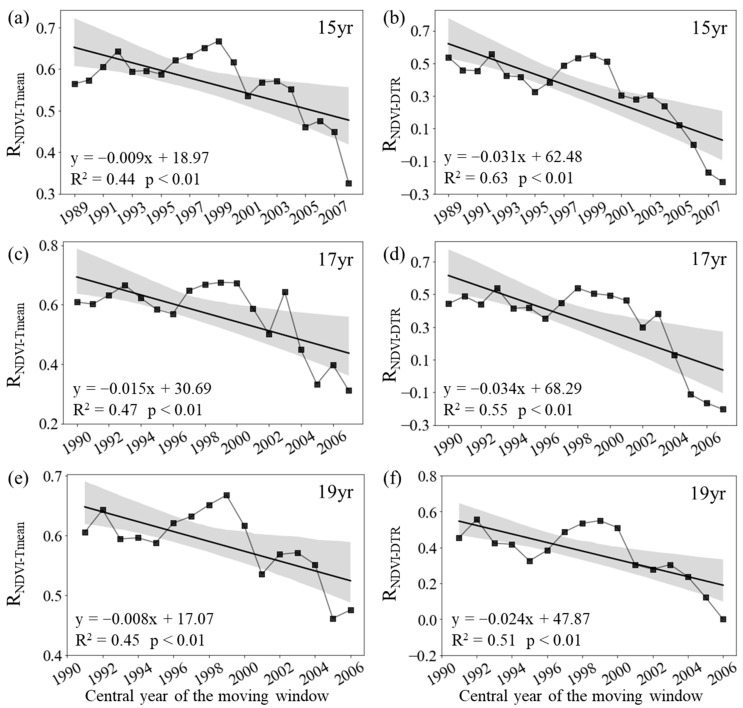
Variations in the sliding partial correlation coefficients between growing season mean NDVI and daily mean temperature ((**a**,**c**,**e**), R_NDVI−Tmean_) and diurnal temperature range ((**b**,**d**,**f**), R_NDVI−DTR_) after applying 15-year (**a**,**b**), 17-year (**c**,**d**), and 19-year (**e**,**f**) moving windows. The black lines represent the inter-annual change trend in the partial correlation coefficients. R^2^ represents the coefficient of determination. Shading denotes 95% prediction intervals.

For the entire period of 1982–2015, the inter-annual variation in the growing season (April to October, GS) NDVI across the boreal regions of the northern hemisphere was significantly correlated with the corresponding GS T_mean_ (R_NDVI−Tmean_ = 0.45, *p* < 0.01), but not with the corresponding GS DTR (R = 0.37, *p* = 0.07). However, the partial correlation coefficients between the GS averaged NDVI and mean daily temperature showed a substantial temporal trend over the past 34 years across these boreal regions (Figure 1). The R_NDVI−Tmean_ was about 0.61 (*p* < 0.05) for the period of 1982–1998 and then generally decreased to about 0.31 (*p* > 0.05) for the period of 1999–2015 (Figure 1c). Similarly, the R_NDVI−DTR_ was about 0.44 (*p* > 0.05) for the period of 1982–1998 and then decreased to about −0.20 (*p* > 0.05) for the period of 1999–2015 (Figure 1d).

Specifically, the R_NDVI−Tmean_ decreased significantly at rates of −0.09/10a (*p* < 0.01, R^2^ = 0.44), −0.15/10a (*p* < 0.01, R^2^ = 0.47), and −0.08/10a (*p* < 0.01, R^2^ = 0.45) with the 15-year, 17-year, and 19-year moving windows, respectively (Figure 1a,c,d). In addition, the R_NDVI−DTR_ also decreased significantly at rates of −0.31/10a (*p* < 0.01, R^2^ = 0.63), −0.34/10a (*p* < 0.01, R^2^ = 0.47), and −0.24/10a (*p* < 0.01, R^2^ = 0.45) with the 15-year, 17-year, and 19-year moving windows, respectively (Figure 1b,d,f). Overall, both the R_NDVI−Tmean_ and R_NDVI−DTR_ showed a weakening trend throughout the last 34 years across the boreal regions.

#### 3.1.2. Intra-Annual Changes in R_NDVI−Tmean_ and R_NDVI−DTR_

**Figure 2:**
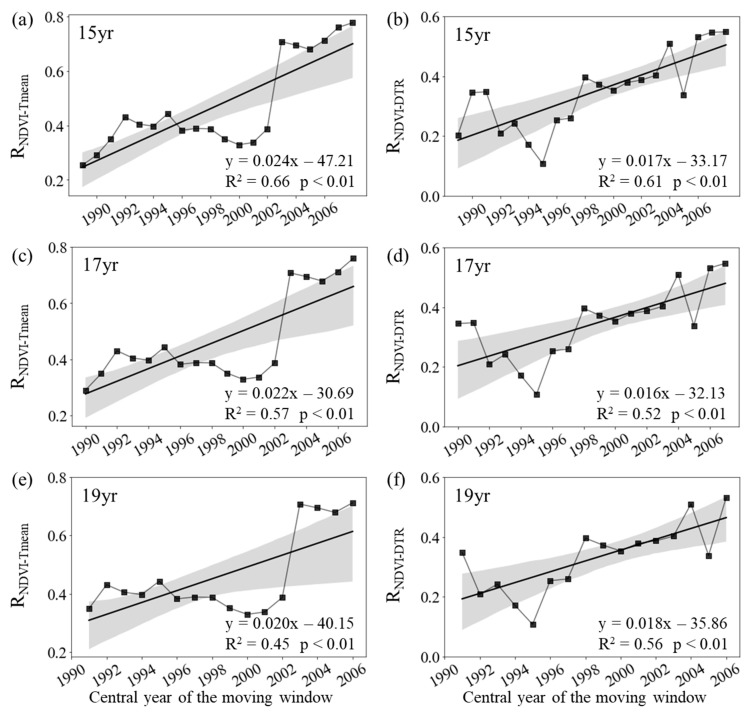
Variations in the sliding partial correlation coefficients between spring mean NDVI and daily mean temperature ((**a**), R_NDVI−Tmean_) and diurnal temperature range ((**b**), R_NDVI−DTR_) after applying 15-year (**a**,**b**), 17-year (**c**,**d**), and 19-year (**e**,**f**) moving windows. The black lines represent the inter-annual change trend in partial correlation coefficients. R^2^ represents the coefficient of determination. Shading denotes 95% prediction intervals.

Both the R_NDVI−Tmean_ and R_NDVI−DTR_ showed distinct patterns among spring, summer, and autumn across the boreal regions. During spring, the annual variation in the mean NDVI for the entire period of 1982–2015 was significantly correlated with the corresponding T_mean_ (R_NDVI−Tmean_ = 0.50, *p* < 0.01) and corresponding DTR (R_NDVI−DTR_ = 0.51, *p* < 0.01). The R_NDVI−Tmean_ was about 0.29 (*p* > 0.05) for the period of 1982–1998 and then generally increased to about 0.76 (*p* < 0.01) for the final window (1999–2015) (Figure 2c). Similarly, we also found that the partial correlation coefficient between the spring mean NDVI and DTR significantly increased from 0.34 (*p* > 0.05) in the first half of the period (1982–1998) to about 0.55 (*p* < 0.01) in the second half (1999–2015) (Figure 2d).

The R_NDVI−Tmean_ for spring increased significantly at rates of 0.24/10a (*p* < 0.01, R^2^ = 0.66), 0.22/10a (*p* < 0.01, R^2^ = 0.57), and 0.20/10a (*p* < 0.01, R^2^ = 0.45) with the 15-year, 17-year, and 19-year moving windows, respectively (Figure 2a,c,d). In addition, the R_NDVI−DTR_ also increased significantly at rates of 0.17/10a (*p* < 0.01, R^2^ = 0.61), 0.16/10a (*p* < 0.01, R^2^ = 0.52), and 0.18/10a (*p* < 0.01, R^2^ = 0.56) with the 15-year, 17-year, and 19-year moving windows, respectively (Figure 1b,d,f). Overall, both the R_NDVI−Tmean_ and R_NDVI−DTR_ showed a strengthening trend over the last 34 years across the boreal regions.

During summer, the annual variation in the mean NDVI for the entire period of 1982–2015 was significantly correlated with the corresponding T_mean_ (R_NDVI−Tmean_ = 0.77, *p* < 0.01), but not with the corresponding DTR (R_NDVI−DTR_ = 0.19, *p* = 0.30). The R_NDVI−Tmean_ was about 0.29 (*p* > 0.05) for the period of 1982–1998 and then generally decreased to about 0.76 (*p* < 0.01) for the final window (1999–2015). Similarly, the summer R_NDVI-DTR_ decreased from 0.10 (*p* > 0.05) in the first half of the period (1982–1998) to about −0.13 (*p* > 0.05) in the second half (1999–2015) (Figure 2d).

**Figure 3:**
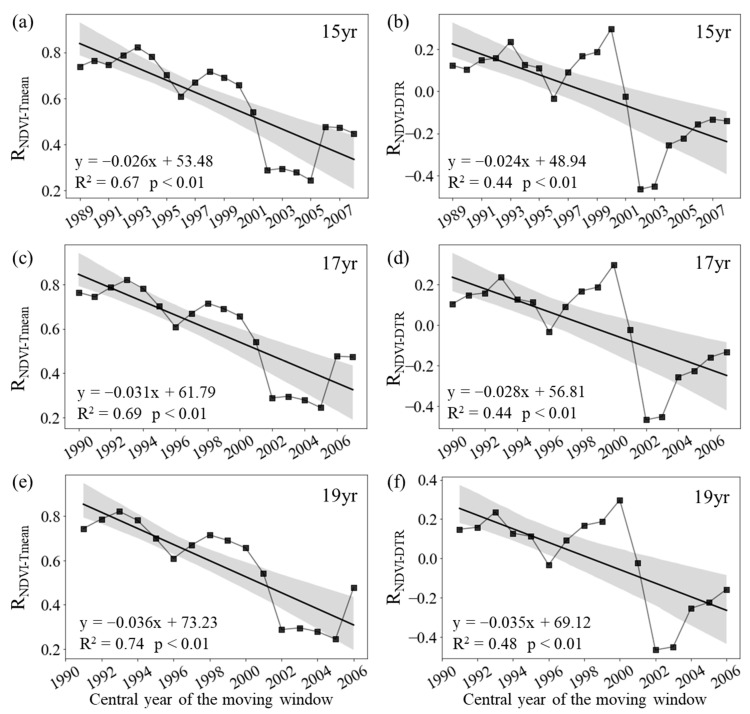
Variations in the sliding partial correlation coefficients between summer mean NDVI and daily mean temperature ((**a**), R_NDVI−Tmean_) and diurnal temperature range ((**b**), R_NDVI−DTR_) after applying 15-year (**a**,**b**), 17-year (**c**,**d**), and 19-year (**e**,**f**) moving windows. The black lines represent the inter-annual change trend in the partial correlation coefficients. R^2^ represents the coefficient of determination. Shading denotes 95% prediction intervals.

Figure 3 shows that the R_NDVI−Tmean_ for summer decreased significantly at rates of −0.26/10a (*p* < 0.01, R^2^ = 0.67), −0.22/10a (*p* < 0.01, R^2^ = 0.69), and −0.36/10a (*p* < 0.01, R^2^ = 0.74) with the 15-year, 17-year, and 19-year moving windows, respectively (Figure 3a,c,d). In addition, the R_NDVI−DTR_ also decreased significantly at rates of −0.24/10a (*p* < 0.01, R^2^ = 0.44), −0.28/10a (*p* < 0.01, R^2^ = 0.44), and −0.35/10a (*p* < 0.01, R^2^ = 0.48) with the 15-year, 17-year, and 19-year moving windows, respectively (Figure 1b,d,f). Therefore, both the summer R_NDVI−Tmean_ and R_NDVI−DTR_ showed a weakening trend throughout the last 34 years across the boreal regions.

**Figure 4:**
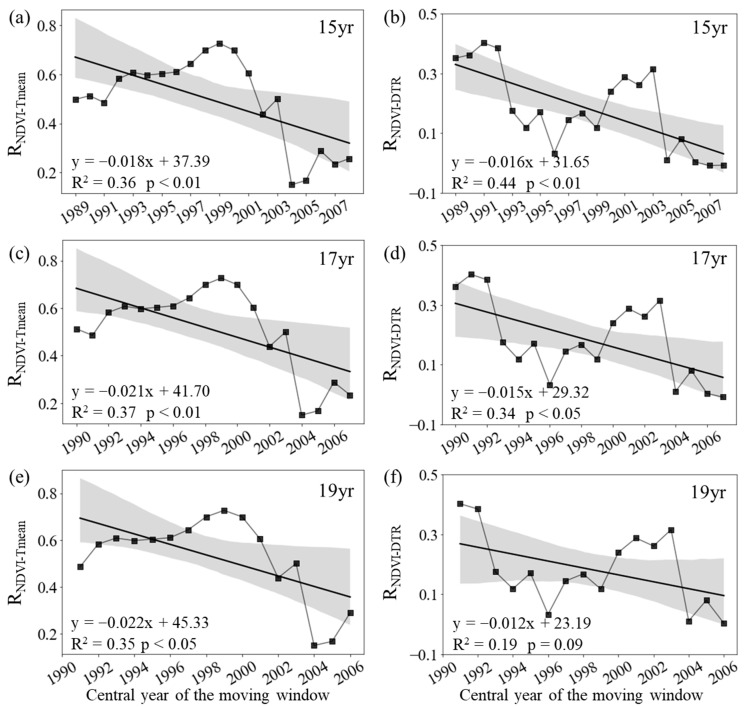
Variations in the sliding partial correlation coefficients between autumn mean NDVI and daily mean temperature ((**a**,**c**,**e**), R_NDVI−Tmean_) and diurnal temperature range ((**b**,**d**,**f**), R_NDVI−DTR_) after applying 15-year (**a**,**b**), 17-year (**c**,**d**), and 19-year (**e**,**f**) moving windows. The black lines represent the inter-annual change trend in the partial correlation coefficients. R^2^ represents the coefficient of determination. Shading denotes 95% prediction intervals.

During autumn, the annual variation in the mean NDVI for the entire period of 1982–2015 was significantly correlated with the corresponding T_mean_ (R_NDVI−Tmean_ = 0.38, *p* < 0.05), but not with the corresponding DTR (R_NDVI−DTR_ = 0.15, *p* > 0.05). The autumn R_NDVI−Tmean_ was about 0.51 (*p* < 0.05) for the period of 1982–1998 and then generally decreased to about 0.24 (*p* > 0.05) for the period of 1999–2015 (Figure 4c). Similarly, the R_NDVI−DTR_ was about 0.10 (*p* > 0.05) for the period of 1982–1998 and then decreased to about −0.13 (*p* > 0.05) for the period of 1999–2015 (Figure 4d).

We also found that both the autumn R_NDVI−Tmean_ and R_NDVI−DTR_ showed a weakening trend over the last 34 years across the boreal regions. Figure 4 shows that the autumn R_NDVI−Tmean_ decreased significantly at rates of −0.18/10a (*p* < 0.01, R^2^ = 0.36), −0.21/10a (*p* < 0.01, R^2^ = 0.37), and −0.22/10a (*p* < 0.05, R^2^ = 0.35) with the 15-year, 17-year, and 19-year moving windows, respectively (Figure 4a,c,d). In addition, the R_NDVI−DTR_ also decreased significantly at rates of −0.16/10a (*p* < 0.01, R^2^ = 0.44) and −0.15/10a (*p* < 0.05, R^2^ = 0.34) with the 15-year and 17-year moving windows, respectively (Figure 4b,d).

### 3.2. Spatial Patterns of the Trends in the Correlations between Vegetation Activity and Temperature Factors

#### 3.2.1. Inter-Annual Patterns of R_NDVI−Tmean_ and R_NDVI−DTR_

**Figure 5:**
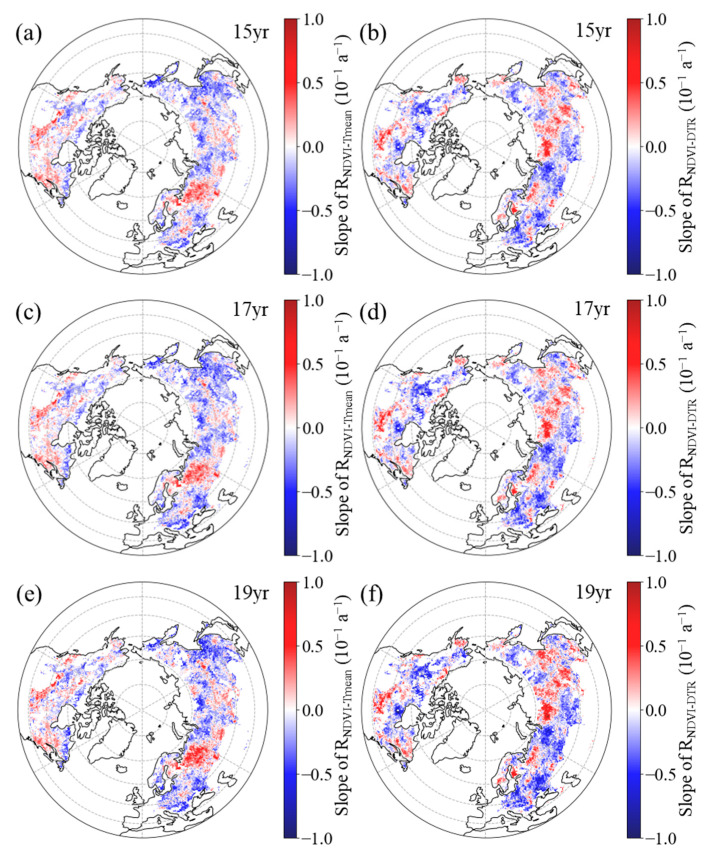
Spatial distribution of the temporal trend of partial correlation coefficient between growing season mean NDVI and daily mean temperature ((**a**,**c**,**e**), R_NDVI−Tmean_) and diurnal temperature range ((**b**,**d**,**f**), R_NDVI−DTR_) during 1982–2015 after applying 15-year (**a**,**b**), 17-year (**c**,**d**), and 19-year (**e**,**f**) moving windows.

Our results indicate that the spatial pattern of the temporal dynamics in the partial correlation coefficients between the NDVI and both the T_mean_ and DTR exhibits a high level of consistency when using three different sliding windows. For the 15-year moving window, in most areas (59.8%), the R_NDVI−Tmean_ for the GS showed a downward trend, with a statistical significance (*p* < 0.05) for 41.2% of the boreal regions (Figure 5a, Table 1). Similarly, in most areas (59.1% of boreal regions), the R_NDVI−DTR_ for the GS showed a downward trend, with a statistical significance (*p* < 0.05) for 40.4% of the boreal regions (Figure 5b, Table 1). For the 17-year moving window, in most areas (62.4%), the R_NDVI−Tmean_ for the GS showed a downward trend, with a statistical significance (*p* < 0.05) for 44.4% of the boreal regions (Figure 5c, Table 1). Similarly, in most areas (59.1% of boreal regions), the R_NDVI−DTR_ for the GS showed a downward trend, with a statistical significance (*p* < 0.05) for 41.2% of the boreal regions (Figure 5d, Table 1). For the 19-year moving window, in most areas (60.0%), the R_NDVI−Tmean_ for the GS showed a downward trend, with a statistical significance (*p* < 0.05) for 40.2% of the boreal regions (Figure 5e, Table 1). Similarly, in most areas (58.6% of boreal regions), the R_NDVI−DTR_ for the GS showed a downward trend, with a statistical significance (*p* < 0.05) for 39.0% of the boreal regions (Figure 5f, Table 1).

Overall, regarding the R_NDVI−Tmean_, the pixels with a significant decreasing trend were primarily distributed in eastern Eurasia and the high latitudes of North America (Figure 5a,c,e). However, the regions where the R_NDVI−Tmean_ exhibited a significant upward trend were concentrated in the southern region of North America and eastern region of Europe (Figure 5a,c,e). Moreover, the R_NDVI−DTR_ pixels with significant decreasing trend were mainly distributed in western Eurasia and the northwestern part of North America (Figure 5b,d,f). In contrast, the regions where the R_NDVI−DTR_ exhibited a significant upward trend were concentrated in the southern region of North America and eastern region of Eurasia (Figure 5b,d,f).

#### 3.2.2. Intra-Annual Patterns of R_NDVI−Tmean_ and R_NDVI−DTR_

**Figure 6:**
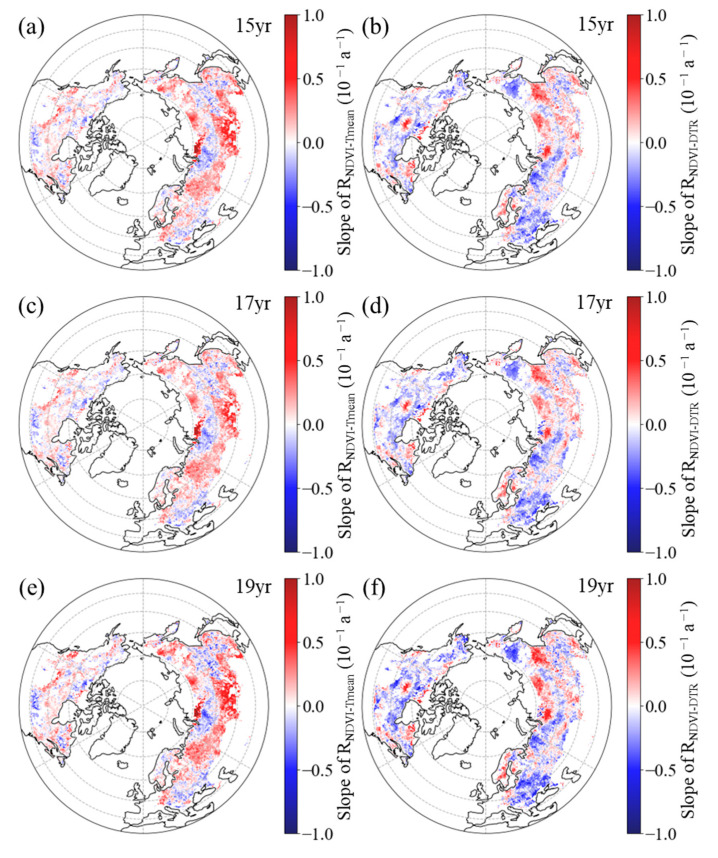
Spatial distribution of the temporal trend of partial correlation coefficient between spring NDVI and daily mean temperature ((**a**,**c**,**e**), R_NDVI−Tmean_) and diurnal temperature range ((**b**,**d**,**f**), R_NDVI−DTR_) for the period 1982–2015 after applying 15-year (**a**,**b**), 17-year (**c**,**d**), and 19-year (**e**,**f**) moving windows.

In spring, the R_NDVI−Tmean_ exhibited a positive temporal trend in 63.3%, 61.7%, and 61.8% of the boreal regions for the 15-year, 17-year, and 19-year moving windows, respectively (Figure 6a,c,e, Table 2). Within the boreal regions, 45.7%, 43.0%, and 42.0% of the pixels showed a significant positive trend for the corresponding moving windows, mainly distributed across the southern regions of Eurasia and central part of North America (Figure 6a,c,e). For the 15-year, 17-year, and 19-year moving windows, negative trends of the R_NDVI−Tmean_ were found in the remaining boreal regions, comprising 36.7%, 38.3%, and 38.2%, respectively (Figure 6a,c,e, Table 2). Among these regions, the trend was found to be significant (*p* < 0.05) in 22.1%, 23.2%, and 22.1% of the areas, respectively, with these pixels primarily being located in the central and eastern parts of Eurasia and northern regions of North America (Figure 6a,c,e).

However, in most areas (53.7%, 53.9%, and 55.3% of the boreal regions for the 15-year, 17-year, and 19-year moving windows, respectively), the spring R_NDVI−DTR_ had a downward trend (Figure 6b,d,f, Table 2). Specifically, for the 15-year, 17-year, and 19-year moving windows, this downward trend was found to be statistically significant (*p* < 0.05) in 34.0%, 35.0%, and 34.5% of the boreal regions, respectively, with these pixels primarily being located in the central and western parts of Eurasia and northern regions of North America (Figure 6b,d,f). Positive trends of the R_NDVI−DTR_ were found in the other 46.3%, 46.1%, and 44.7% of the boreal regions for the 15-year, 17-year, and 19-year moving windows, respectively (Figure 6b,d,f, Table 2). Additionally, across the boreal regions, 28.07%, 28.0%, and 25.3% of the pixels showed a significant positive trend for the corresponding moving windows, with these pixels mainly being distributed across the eastern parts of Eurasia and northeastern regions of North America (Figure 6b,d,f).

**Figure 7:**
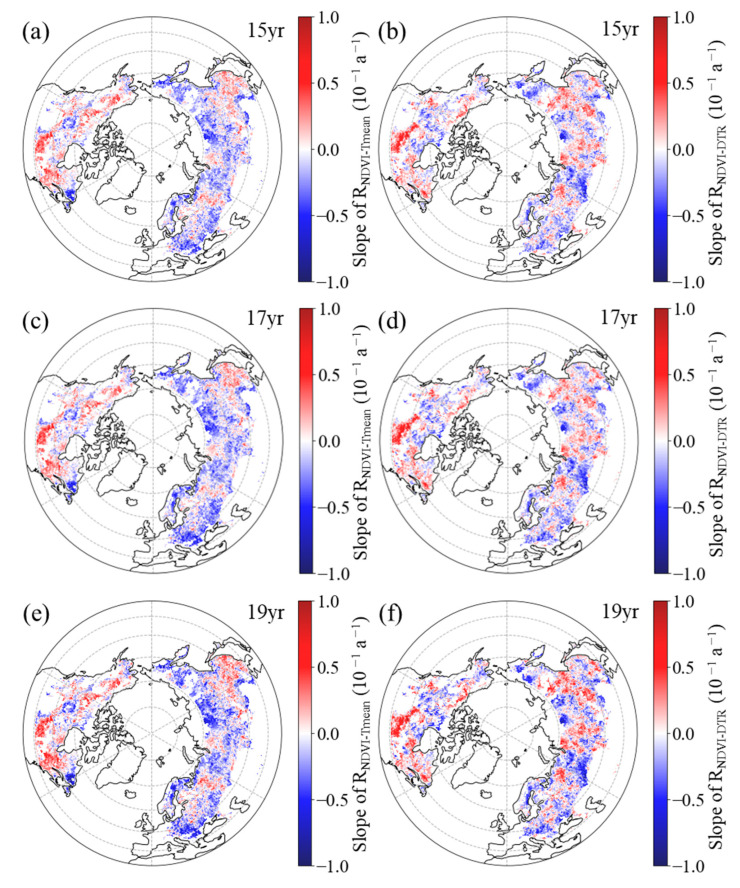
Spatial distribution of the temporal trend of partial correlation coefficient between summer NDVI and daily mean temperature ((**a**,**c**,**e**), R_NDVI−Tmean_) and diurnal temperature range ((**b**,**d**,**f**), R_NDVI−DTR_) during 1982–2015 after applying 15-year (**a**,**b**), 17-year (**c**,**d**), and 19-year (**e**,**f**) moving windows.

In summer, the R_NDVI−Tmean_ exhibited a negative temporal trend in 60.2%, 62.4%, and 60.2% of the boreal regions for the 15-year, 17-year, and 19-year moving windows, respectively (Figure 7a,c,e, Table 3). Additionally, across the boreal regions, 41.7%, 45.2%, and 40.6% of the pixels showed a significant negative trend for the corresponding moving windows, with these pixels primarily being located in the central and western parts of Eurasia and northeastern regions of North America (Figure 7a,c,e). For the 15-year, 17-year, and 19-year moving windows, positive trends of the R_NDVI−Tmean_ were found in the remaining boreal regions, comprising 39.8%, 37.6%, and 39.8%, respectively (Figure 7a,c,e, Table 3). Among these regions, the positive trend was found to be significant (*p* < 0.05) in 23.4%, 22.0%, and 22.2% of the areas, respectively, with these pixels mainly being located in the eastern parts of Eurasia and southern regions of North America (Figure 7a,c,e).

Similarly, in most areas (54.3%, 54.8%, and 53.5% of the boreal regions for the 15-year, 17-year, and 19-year moving windows, respectively), the spring R_NDVI−DTR_ had a downward trend (Figure 7b,d,f, Table 3). Within the boreal regions, 36.0%, 36.9%, and 34.7% of the pixels showed a significant downward trend for the corresponding moving windows, mainly being located in the western regions of Eurasia and northwestern regions of North America (Figure 7b,d,f). Positive trends of the R_NDVI−DTR_ were found in the other 45.7%, 45.2%, and 46.5% of the boreal regions for the 15-year, 17-year, and 19-year moving windows, respectively (Figure 7b,d,f, Table 3). Additionally, across the boreal regions, 28.8%, 28.7%, and 28.2% of the pixels showed a significant positive trend for the corresponding moving windows, with these pixels mainly being located in the eastern regions of Eurasia and southern regions of North America (Figure 7b,d,f).

**Figure 8:**
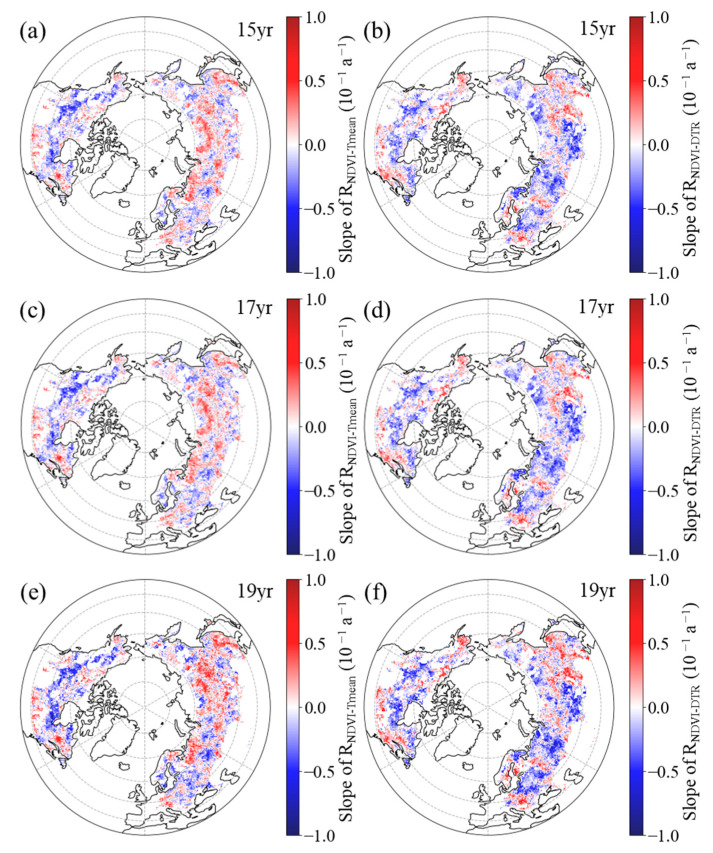
Spatial distribution of the temporal trend of partial correlation coefficient between autumn NDVI and daily mean temperature ((**a**,**c**,**e**), R_NDVI−Tmean_) and diurnal temperature range ((**b**,**d**,**f**), R_NDVI−DTR_) during 1982–2015 after applying 15-year (**a**,**b**), 17-year (**c**,**d**), and 19-year (**e**,**f**) moving windows.

In autumn, the R_NDVI−Tmean_ exhibited a negative temporal trend in 50.6%, 51.3%, and 52.2% of the boreal regions for the 15-year, 17-year, and 19-year moving windows, respectively (Figure 8a,c,e, Table 4). Additionally, across the boreal regions, 31.5%, 32.3%, and 32.5% of the pixels showed a significant negative trend for the corresponding moving windows, with these pixels mainly being located in the western regions of Eurasia and central regions of North America (Figure 8a,c,e). For the 15-year, 17-year, and 19-year moving windows, positive trends of the R_NDVI−Tmean_ were found in the remaining boreal regions, comprising 49.4%, 48.8%, and 47.8%, respectively (Figure 8a,c,e, Table 4). Among these regions, the positive trend was found to be significant (*p* < 0.05) in 30.4%, 30.0%, and 29.4% of the areas, respectively, with these pixels primarily being located in the central and eastern regions of Eurasia and southern regions of North America (Figure 8a,c,e).

Similarly, in most areas (60.1%, 60.2%, and 57.5% of the boreal regions for the 15-year, 17-year, and 19-year moving windows, respectively), the spring R_NDVI−DTR_ had a downward trend (Figure 8b,d,f, Table 4). Within the boreal regions, 40.8%, 40.9%, and 37.2% of the pixels showed a significant downward trend for the corresponding moving windows, mainly being located in the central and western regions of Eurasia and northern regions of North America (Figure 8b,d,f). Positive trends of the R_NDVI−DTR_ were found in the other 39.9%, 39.8%, and 42.5% of the boreal regions for the 15-year, 17-year, and 19-year moving windows, respectively (Figure 8b,d,f, Table 4). Additionally, across the boreal regions, 23.0%, 23.5%, and 24.4% of the pixels showed a significant positive trend for the corresponding moving windows, with these pixels mainly being located in the eastern regions of Eurasia and southern regions of North America (Figure 8b,d,f).

## 4. Discussion

Our results show considerable temporal trends in the partial correlation coefficients between the GS NDVI and daily mean temperature across the boreal regions over the last 34 years. This variability indicates a weakening relationship between the interannual temperature variability and boreal vegetation activity. Varying responses of vegetation activity to global warming can be also found in the results of previous studies on high-latitude or high-altitude areas [5,23]. For example, Cong et al. [23] found that the relationship between the summer NDVI and air temperature for an alpine meadow across the Tibetan Plateau showed a decreasing trend. Piao et al. [5] found that, in the northern hemisphere, the positive influence of the growing season air temperature on the vegetation growth exhibited a weakening trend over the last three decades. Overall, our finding of an interannual change in the response of boreal vegetation activity to air temperature is either a verification of, or a supplement to, previous studies at different scales.

Here, we also analyzed the temporal variation in the boreal vegetation response to air temperature at the seasonal scale, which has been neglected in previous studies. We found a significant weakening trend in the relationship between the NDVI and T_mean_, mainly in the summer and autumn seasons. This timing means that the weakening in the influence of the air temperature on the vegetation activity during the summer and autumn seasons dominates the overall weakening in the correlation during the GS. In contrast, during the spring, we found an increasing trend in the relationship between the NDVI and temperature, suggesting that, in the spring, the relationship between temperature changes and vegetation growth may have become more closely linked.

The reasons for these changes in the vegetation activity response to temperature changes are still difficult to verify due to the complexity of and variability in the factors involved. Possible explanations for the weakening trend in the vegetation response to temperature during summer are that, under the background of global warming, the temperature in the region may have approached the optimal temperature or physiological and ecological threshold of some plants, or that the vegetation has gradually adapted to the warming environment. Piao et al. [5] suggested that the weakening relationship between vegetation activity and temperature changes can be attributed to the increasing frequency of drought in the northern hemisphere. Qin et al. [24] supported this idea and found that the relationship between vegetation activity and temperature during warm years exhibited a significant decreasing trend. However, the temperature rise in spring has led to an earlier start to the growing season for vegetation in the northern high latitudes [25]. For instance, Piao et al. [25] found an earlier onset of spring (0.16 days yr^−1^) over the last two decades across the northern hemisphere. The continuous increase in the vegetation GS caused by global warming is almost certainly one reason for the growing enhancement of the vegetation response to temperature.

In addition, we found that the effect of the GS diurnal temperature range on the vegetation activity also showed a weakening trend. This finding has important implications for refining the understanding of the effects of diurnal asymmetric warming on terrestrial ecosystems [12,24]. For example, Zhao et al. [12] found that the strength of the relationship between the greenness of high-latitude vegetation and daytime warming exhibited a weakening trend at the inter-annual scale. However, in most of the boreal regions of North America, there was an increasing trend in the correlation between nighttime warming and vegetation activity [12].

Multiple uncertainties still remain in understanding the temporal dynamics of the boreal vegetation activity response to temperature. A few indirect factors, such as fire disturbance [3,26], insect damage [27], spring frosts, land use and land management changes [28,29], and changes in nutrient use efficiency also have the potential to weaken this observed relationship between vegetation activity and temperature change. These factors could also explain some of the differences in the spatial patterns of the temporal trends of the NDVI–temperature correlations.

## 5. Conclusions

Our study analyzed the temporal trends in the boreal vegetation activity response to mean daily temperature and diurnal temperature range temperature changes at the regional and seasonal scales over the past 34 years. We found that the GS R_NDVI−Tmean_ showed a significant decreasing (−0.15/10a) trend throughout the last three decades across the boreal regions with the 17-year moving window, which was mainly due to the decreasing R_NDVI−Tmean_ in summer (−0.31/10a) and autumn (−0.21/10a). In contrast, the R_NDVI-Tmean_ for spring increased significantly at the rate of 0.22/10a, suggesting that the relationship between mean daily temperature changes and vegetation activity may have become more closely linked in the spring. Similar to the R_NDVI−Tmean_, the GS R_NDVI−DTR_ also showed a significant decreasing (−0.34/10a) trend over the last three decades across the boreal regions with the 17-year moving window. This was mainly due to the decreasing R_NDVI−DTR_ in summer (−0.28/10a) and autumn (−0.12/10a). In terms of spatial patterns, areas showing a significantly negative trend in the GS R_NDVI−Tmean_ and R_NDVI−DTR_ accounted for approximately 44.4% and 41.2% of the boreal regions with the 17-year moving window, respectively. In contrast, areas that exhibited a significant increasing trend in the GS R_NDVI−Tmean_ and R_NDVI−DTR_ accounted for only approximately 22.3% and 25.8% of the boreal regions with the 17-year moving window, respectively. At the seasonal scale, the area showing a significantly negative trend in the partial correlation coefficient between the vegetation activity and temperature factors was bigger than the area with a significant positive trend for both summer and autumn. In spring, however, the area with a significant upward trend in the R_NDVI−Tmean_ and R_NDVI−DTR_ was greater than that with a significant downward trend.

## Figures and Tables

**Table 1:** Percentages of the area with a positive or negative temporal trend of the partial correlation coefficient between growing season mean NDVI and daily mean temperature (R_NDVI−Tmean_) and diurnal temperature range (R_NDVI-DTR_) during 1982–2015. The values within parentheses represent the proportion that has passed the statistical significance test (*p* < 0.05).

	R_NDVI−Tmean_		R_NDVI−DTR_	
	Positive	Negative	Positive	Negative
15-year	40.2% (24.0%)	59.8% (41.2%)	40.9% (24.9%)	59.1% (40.4%)
17-year	37.6% (22.3%)	62.4% (44.4%)	40.9% (25.8%)	59.1% (41.2%)
19-year	40.0% (22.7%)	60.0% (40.2%)	41.4% (24.8%)	58.6% (39.0%)

**Table 2:** Percentages of the area with a positive or negative temporal trend of the partial correlation coefficient between spring NDVI and daily mean temperature (R_NDVI−Tmean_) and diurnal temperature range (R_NDVI−DTR_) during 1982–2015. The values within parentheses represent the proportion that has passed the statistical significance test (*p* < 0.05).

	R_NDVI−Tmean_		R_NDVI−DTR_	
	Positive	Negative	Positive	Negative
15-year	63.3% (45.7%)	36.7% (22.1%)	46.3% (28.0%)	53.7% (34.0%)
17-year	61.7% (43.0%)	38.3% (23.2%)	46.1% (28.0%)	53.9% (35.0%)
19-year	61.8% (42.0%)	38.2% (22.1%)	44.7% (25.3%)	55.3% (34.5%)

**Table 3:** Percentages of the area with a positive or negative temporal trend of the partial correlation coefficient between summer NDVI and daily mean temperature (R_NDVI−Tmean_) and diurnal temperature range (R_NDVI−DTR_) during 1982–2015. The values within parentheses represent the proportion that has passed the statistical significance test (*p* < 0.05).

	R_NDVI−Tmean_		R_NDVI−DTR_	
	Positive	Negative	Positive	Negative
15-year	39.8% (23.4%)	60.2% (41.7%)	45.7% (28.8%)	54.3% (36.0%)
17-year	37.6% (22.0%)	62.4% (45.2%)	45.2% (28.7%)	54.8% (36.9%)
19-year	39.8% (22.2%)	60.2% (40.6%)	46.5% (28.2%)	53.5% (34.7%)

**Table 4:** Percentages of the area with a positive or negative temporal trend of the partial correlation coefficient between autumn NDVI and daily mean temperature (R_NDVI−Tmean_) and diurnal temperature range (R_NDVI−DTR_) during 1982–2015. The values within parentheses represent the proportion that has passed the statistical significance test (*p* < 0.05).

	R_NDVI−Tmean_		R_NDVI−DTR_	
	Positive	Negative	Positive	Negative
15-year	49.4% (30.4%)	50.6% (31.5%)	39.9% (23.0%)	60.1% (40.8%)
17-year	48.8% (30.0%)	51.3% (32.3%)	39.8% (23.5%)	60.2% (40.9%)
19-year	47.8% (29.4%)	52.2% (32.5%)	42.5% (24.4%)	57.5% (37.2%)

## Data Availability

The data used in the present work have been listed in the Data Sources.
